# A novel human peristalsis-inspired 3D-printed gastroduodenal simulator to evaluate intragastric/duodenal metabolic devices: a proof-of-concept study

**DOI:** 10.1186/s12967-022-03357-z

**Published:** 2022-04-01

**Authors:** Jinhee Kwon, Chang Seok Bang, Sung Ock Kim, Do Hyun Park

**Affiliations:** 1 Division of Gastroenterology, Department of Internal Medicine, University of Ulsan College of Medicine, Asan Medical Center, Seoul, Republic of Korea; 2 Department of Medical Science, Asan Medical Institute of Convergence Science and Technology, Asan Medical Center, University of Ulsan College of Medicine, Seoul, Republic of Korea; 3 Department of Internal Medicine, Hallym University Chuncheon Sacred Heart Hospital, Chuncheon, Republic of Korea; 4 Department of Radiology, University of Ulsan College of Medicine, Asan Medical Center, Seoul, Republic of Korea; 5 Digestive Diseases Research Center, Department of Internal Medicine, University of Ulsan College of Medicine, Asan Medical Center, 88, Olympic-ro 43-gil, Songpa-gu, Seoul, 05505 Republic of Korea

**Keywords:** Obesity, Gastric emptying, Endoscopic bariatric and metabolic therapies, 3D-printed gastroduodenal simulator

To the Editor,

Endoscopic bariatric and metabolic therapies (EBMT) is an attractive alternative to medical treatment and bariatric surgery owing to the efficacy and minimal invasiveness. Delay in gastric emptying or gastric retention with filling of the ingested food in stomach triggers satiation, and rapid gastric emptying is related to obesity [1, 2]. Therefore, delayed gastric emptying should be evaluated with an intragastric/duodenal device for EBMT to assess its performance in inducing weight loss in patients with obesity. However, no quantitative comparative results on the performance of delayed gastric emptying in intragastric balloons (IGBs), and gastro-duodenal flow restrictors (G-DFR) have been reported in a pre-clinical study. The aim of the present study was to introduce the 3D-printed gastroduodenal simulator that mimics human peristalsis as a virtual testing system for the quantitative measurement of delayed gastric emptying in novel intragastric/duodenal EBMT devices before animal trials can be conducted. We also planned a pilot animal study whether this novel device with substantial delayed gastric emptying in experimental porcine group may affect relative weight loss compared with control porcine group.

The geometry and dimensions of the gastroduodenal simulator were designed to match the shape of the human stomach, based on computed tomography gastrography images of a healthy volunteer from a previous study [3]. The simulator was fabricated using 3D-printed molds (Omg SLA; Xiamen, China), elastomer (Ecoflex 00-30; Smooth-on, Inc., USA), and silicone adhesive (Sil-Poxy; Smooth-on, Inc.). The simulator comprised four segments (fundus, body, antrum, and pylorus-duodenal bulb), each of which was paired with two actuators (L16-R Miniature Linear Servos; Actuonix, British Columbia, Canada) to contact the stomach body. To simulate peristalsis, eight motors moving in a straight line were mounted to generate motility in these regions. The contraction wave (α), which controls the stroke parameter of peristalsis, was defined as follows: α = diameter in contraction/diameter in relaxation. Each region was arranged with a relative contraction wave (α) of 0.9, 0.6, 0.6, and 0.2, respectively. The frequency parameter of peristalsis was set to twice per minute. Measurements in the simulator with peristalsis were obtained at different stages of drained and residual gastric material to assess gastric emptying volume defined as the partly drained fluid mass collected and retention ratio measured residual markers in the stomach. The normal range of values for gastric retention and gastric emptying after 2 h following a liquid meal were obtained from a prior study of 10 asymptomatic healthy volunteers in which it was defined as 25.9% ± (2 × 12.5%) [4]. This was consistent with the retention ratio of 28.61% we observed in our control experiment using the simulator with peristalsis for 4 h. (Additional file 1: Video S1).

The gastric emptying volume of experimental model occupying G-DFR with 60-mm polytetrafluoroethylene (PTFE) skirt was the least gastric emptying volume suggesting enhanced gastric retention (percentage of emptying volume: 32.4%, retention: 49.2%) compared with IGB (percentage of emptying volume: 47.9%, retention: 37.9%), and G-DFR with a 30-mm PTFE skirt (percentage of emptying volume: 41.7%, retention: 35.0%). (Fig. 1) To clarify the quantitative measurement of delayed gastric emptying with favorable results on G-DFR with 60 mm distal PTFE skirt in this simulator with peristalsis, exploratory porcine study was performed. (Fig. 2A) This pilot porcine study was only evaluated the short-term outcomes (28-day and 42-day follow up) in terms of body weight and G-DFR migration. In the exploratory porcine (Yorkshire pig, 35–40 kg, 2 in experimental and 2 in control group) study, experimental porcine group with a novel G-DFR showed relative weight loss relative to the control group but without statistical significance: − 0.7% vs. 1.4% in 3 days; 5.2% vs. 15.7% in 14 days; 16.3% vs. 32.9% in 28 days; and 25.3% vs. 48.0% in 42 days. (Fig. 2B) No proximal or distal migration of a novel G-DFR was observed in the experimental group. (Additional file 2: Video S2).

**Fig. 1:**
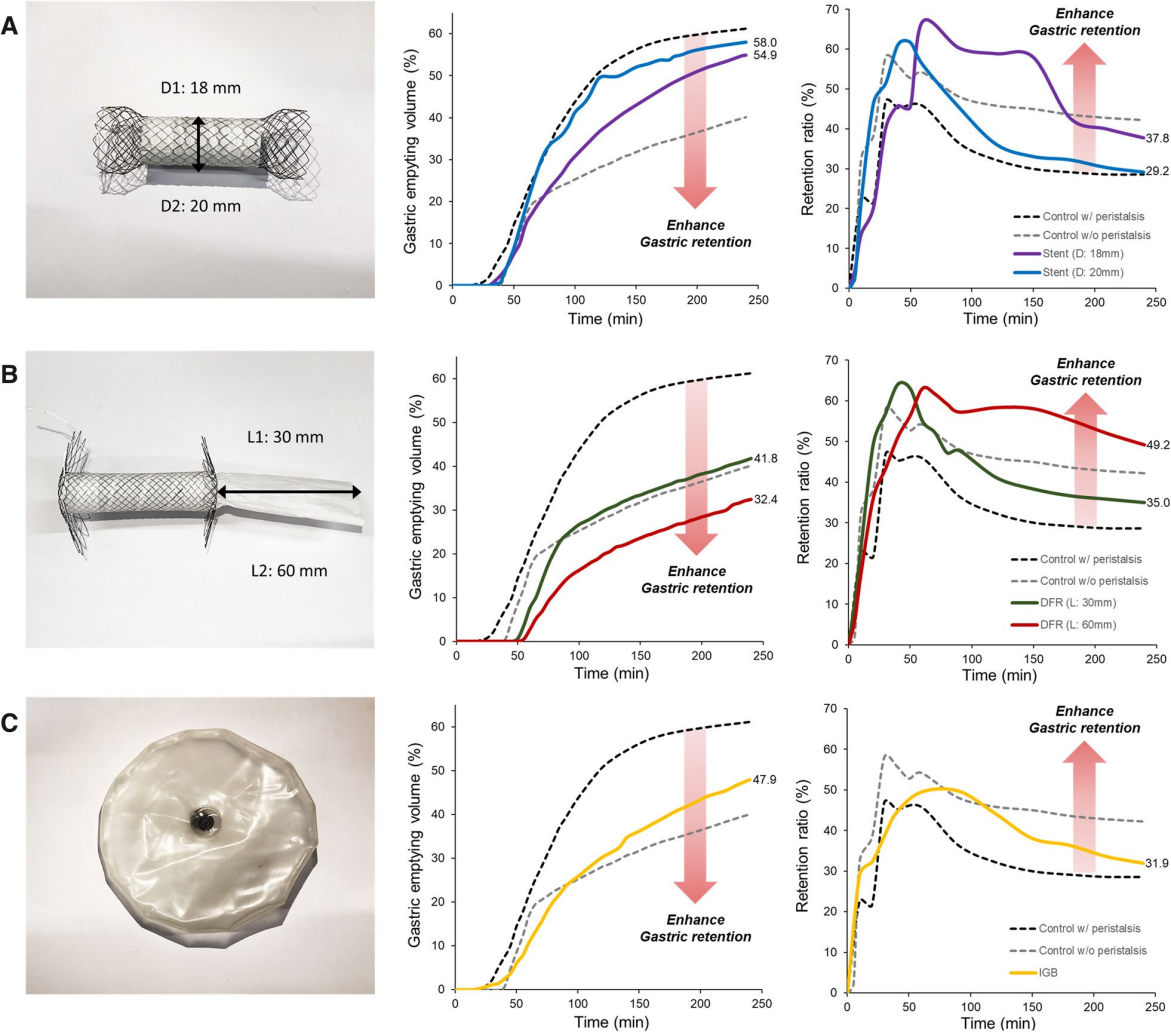
Comparison of the changes in delayed gastric emptying volumes [ml] and percentage retention ratio of radio-opaque markers [%] in the simulator between the control group without intragastric/duodenal metabolic devices (dotted black line—with peristalsis, dotted gray line—without peristalsis as the standard reference model for delayed gastric emptying) and experimental devices including **A** commercial gastroduodenal covered metal stent with an 18 mm (purple line) or 20 mm (blue line) diameter, **B** G-DFR devices with a 30 mm (green line) or 60 mm (red line) PTFE skirt, and **C** an intragastric balloon (yellow line). *D* diameter of a stent, *L* length of PTFE skirt in G-DFR

**Fig. 2:**
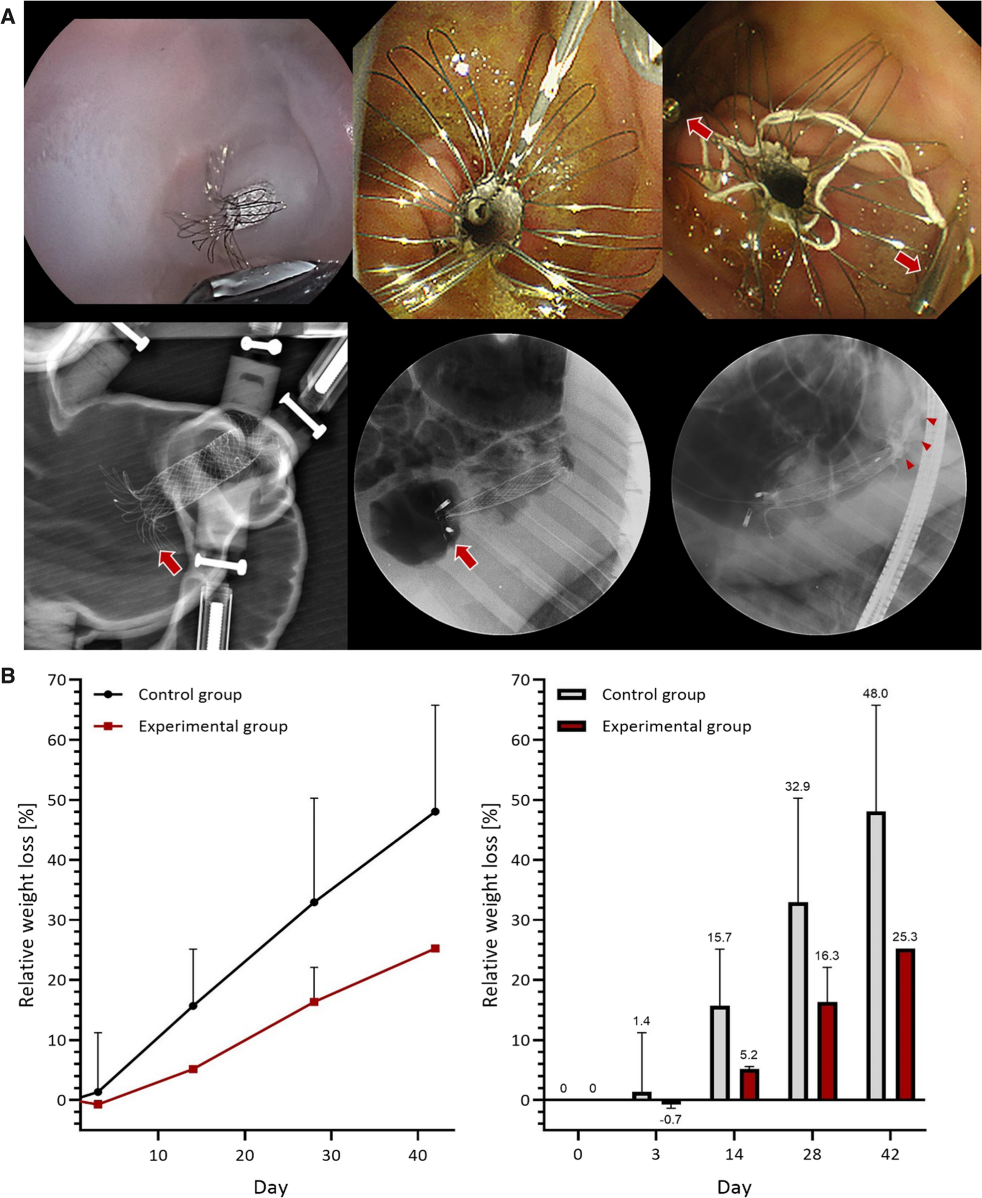
**A** Endoscopic images of the gastric duodenal simulator (upper-left) and a porcine stomach after deployment of the novel gastro-duodenal flow restrictor (G-DFR) (upper-middle). The pig model used two hemoclips (arrowheads) to anchor the proximal flap of the G-DFR (upper-right). Radiographs showing the G-DFR in a simulator with peristalsis (lower-left) and the porcine stomach (lower-middle) after deployment of the novel G-DFR. Injected contrast slowly ran off through a 60-mm distal PTFE skirt of G-DFR (lower-right, arrowheads). **B** Line graphs showing the differences in relative weight loss percentages between the control and experimental porcine groups with the placement of a G-DFR with a 60-mm distal PTFE skirt (left). Bar graphs representing the difference of body weight between the control and the experimental group at each time point (right)

In summary, we could quantitatively define the delayed gastric emptying volume for intragastric and gastroduodenal metabolic devices and evaluate the differences between these devices, which led to a delayed gastric emptying. The promising relative weight loss of our preliminary porcine study may suggest that this 3D-printed peristaltic simulator platform may predict the performance of a novel G-DFR with optimal delayed gastric emptying for EBMT.

## Supplementary Information

**Additional file 1: Video S1**. The methods and results of the experimental investigation in a simulator with peristalsis.

**Additional file 2: Video S2**. The methods and results of the exploratory porcine (Yorkshire pig, 35-40kg, 2 in experimental and 2 in control group) study.

## Data Availability

The datasets used and/or analyzed during the current study are available from the corresponding author on reasonable request.

## Abbreviations

EBMT: Endoscopic bariatric and metabolic therapies
IGB: Intragastric balloon
G-DFR: Gastro-duodenal flow restrictors
PTFE: Polytetrafluoroethylene
